# Preliminary findings and feasibility of mid‐life cognitive and blood‐based biomarker assessments in adults with and without prospectively studied ADHD diagnosis in childhood

**DOI:** 10.1002/alz70857_100519

**Published:** 2025-12-24

**Authors:** Brooke Molina, Douglas Teixeira Leffa, Ann D Cohen, Heidi Kipp, Laura Disarro, Kenneth Connor, Abigail Beattie, Brandy L. Callahan, Tharick A Pascoal, Thomas K Karikari

**Affiliations:** ^1^ University of Pittsburgh, Pittsburgh, PA, USA; ^2^ University of Pittsburgh Alzheimer's Disease Research Center (ADRC), Pittsburgh, PA, USA; ^3^ University of Pittsburgh School of Medicine, Pittsburgh, PA, USA; ^4^ University of Calgary and University of Calgary & Hotchkiss Brain Institute, Calgary, AB, Canada

## Abstract

**Background:**

Attention Deficit Hyperactivity Disorder (ADHD) is a prevalent neurodevelopmental disorder associated with long‐term, impairing symptoms of inattention and/or impulsivity/hyperactivity‐restlessness into adulthood and late‐life. Emerging epidemiological evidence suggests that ADHD is associated with an increased risk for mild cognitive impairment (MCI) and Alzheimer's disease (AD). Furthermore, genetic predisposition to ADHD has been linked to cognitive decline and AD‐related pathophysiology. It is unclear whether AD risk in ADHD is driven by reduced brain resilience to the effects of AD pathophysiology (e.g., amyloid pathology) or shared risk factors that directly affect AD pathology (e.g., cardiovascular health). Pathways to AD have been principally tested in samples free from neurodevelopmental differences, making it impossible to know the applicability of commonly studied AD pathways in vulnerable groups. Moreover, studies of ADHD in AD have been hampered by reliance on electronic health records, which can present biased estimates of ADHD prevalence due to unclear diagnostic accuracy.

**Method:**

To begin addressing these issues, we are conducting cognitive and AD biomarker assessments with 100 participants from the Pittsburgh ADHD Longitudinal Study (PALS), a cohort of individuals with rigorous diagnoses of ADHD in childhood. Participants are in their 40s, with 25/100 assessed to date, 19 ADHD and 6 nonADHD, M_age_=44.5, 28% Black or multiple race, 24% women. We anticipate 50 assessments by 06/25. This research is studying feasibility of procedures in the larger PALS sample and gathering preliminary data about group differences in cognitive functioning pertinent to ADHD and AD risk and blood‐based biomarkers of AD, some of which are known to be altered in preclinical stages of the disease. Example cognitive tests are MoCA and Rey Auditory Verbal Learning Test–Immediate/Recall; example blood‐based biomarkers are plasma Ab42/40, tau (*p*‐tau181, 217), and inflammation‐related markers.

**Result:**

We will report preliminary group comparisons, emphasizing effect sizes given interim study progress.

**Conclusion:**

Understanding the association between rigorous, prospectively diagnosed ADHD and prevalent age‐related diseases, such as AD, is a pressing concern given the increased prevalence of ADHD in adulthood. Our research is beginning to address this need including demonstrating feasibility of measuring cognition and AD biomarkers in midlife adults with ADHD histories.

